# CitGATA7 interact with histone acetyltransferase CitHAG28 to promote citric acid degradation by regulating the glutamine synthetase pathway in citrus

**DOI:** 10.1186/s43897-024-00126-y

**Published:** 2025-02-01

**Authors:** Xiahui Lin, Shaojia Li, Yanna Shi, Yuchen Ma, Yinchun Li, Haohan Tan, Bo Zhang, Changjie Xu, Kunsong Chen

**Affiliations:** 1 College of Agriculture and Biotechnology, Zhejiang University, Zijingang Campus, Hangzhou, 310058 P.R. China; 2 Zhejiang Key Laboratory of Horticultural Crop Quality Improvement, Zhejiang University, Zijingang Campus, Hangzhou, 310058 P.R. China; 3 The State Agriculture Ministry Laboratory of Horticultural Plant Growth, Development and Quality Improvement, Zhejiang University, Zijingang Campus, Hangzhou, 310058 P.R. China

**Keywords:** Citrus, Citric acid, Transcriptional regulation, Histone acetylation, Habitats

## Abstract

Organic acid is a crucial indicator of fruit quality traits. Citric acid, the predominant organic acid in citrus fruit, directly influences its edible quality and economic value. While the transcriptional regulatory mechanisms of citric acid metabolism have been extensively studied, the understanding about the transcriptional and epigenetic co-regulation mechanisms is limited. This study characterized a transcription factor, CitGATA7, which directly binds to and activates the expression of genes associated with the glutamine synthetase pathway regulating citric acid degradation. These genes include the aconitase encoding gene *CitACO3*, the isocitrate dehydrogenase encoding gene *CitIDH1*, and the glutamine synthetase encoding gene *CitGS1*. Furthermore, CitGATA7 physically interacts with the histone acetyltransferase CitHAG28 to enhance histone 3 acetylation levels near the transcription start site of *CitACO3*, *CitIDH1*, and *CitGS1*, thereby increasing their transcription and promoting citric acid degradation. The findings demonstrate that the CitGATA7-CitHAG28 protein complex transcriptionally regulate the expression of the GS pathway genes, i.e., *CitACO3*, *CitIDH1*, and *CitGS1*, via histone acetylation, thus promoting citric acid catabolism. This study establishes a direct link between transcriptional regulation and histone acetylation regarding citric acid metabolism, providing insights for strategies to manipulate organic acid accumulation in fruit.

**Supplementary Information:**

The online version contains supplementary material available at 10.1186/s43897-024-00126-y.

## Core

The transcription factor CitGATA7 functions as a positive regulator of citric acid degradation by targeting genes associated with the glutamine synthetase (GS) pathway, including *CitACO3*, *CitIDH1*, and *CitGS1*. Furthermore, CitGATA7 interacts with the histone acetyltransferase CitHAG28 to enhance histone 3 acetylation levels near the transcription start site of *CitACO3*, *CitIDH1*, and *CitGS1*, thereby synergistically promoting citric acid degradation through activation of GS pathway-associated gene transcription.

## Gene and accession numbers

Gene information utilized in this study is accessible in the Citrus genome database (http://citrus.hzau.edu.cn), with the following accession numbers: *CitACO3* (Ciclev10007338m.g); *CitIDH1* (Ciclev10001314m.g); *CitGSI* (Ciclev10031939m.g); *CitGATA7* (Ciclev10003699m.g); and *CitHAG28* (Ciclev10006418m.g).

## Introduction

Organic acid is metabolite involved in carbohydrate conversion, amino acid biosynthesis, and the maintenance of redox and energy equilibrium (Igamberdiev and Eprintsev 2016). Fruit acidity, a critical component of fruit organoleptic quality, is determined by the organic acid content in fruit (Lado et al. 2018). Generally, organic acids accumulate during fruit growth, and degrade during fruit ripening, a process influenced by various metabolic enzymes, acid transporters, and proton pumps (Ye et al. 2017; Liu et al. 2021; Huang et al. 2023; Lu et al. 2024). This process is also regulated by multiple transcription factors (TFs), including MYB (Hu et al. 2016; Peng et al. 2023), bHLH (Yu et al. 2021), WRKY (Alabd et al. 2023; Wang et al. 2023), and NAC (Li et al. 2017b; Fu et al. 2023).

Citrus fruit possesses a distinctive flavor profile, with the sugar-acid content and ratio serving as the primary determinant of its unique taste. Citric acid constitutes 60%-90% of the total organic acids in citrus fruit, synthesized in the tricarboxylic acid cycle (TCA) within the mitochondrion and predominantly degraded in the cytoplasm (Rouseff et al. 2009). The degradation of citric acid occurs through three main pathways: the ATP-citrate lyase (ACL) pathway, the gamma‑aminobutyric acid (GABA) shunt pathway, and the glutamine synthetase (GS) pathway (Sheng et al. 2017; Li et al. 2020a). In the GS pathway, citric acid is initially catalyzed by aconitase (ACO) and then by isocitrate dehydrogenase (IDH) to produce 2-ketoglutaric acid, which is subsequently converted to glutamate (Etienne et al. 2013). Despite its significance in citric acid degradation, the GS pathway remains understudied.

Histone acetylation, a type of epigenetic modification, regulates gene transcription by altering nucleosome structure stability and chromatin open conformation. This process is reversibly regulated by histone acetyltransferases (HAT) and histone deacetylases (HDAC) (Wellen et al. 2009; Shen et al. 2015). Generally, HAT facilitates chromatin opening, enabling transcription regulators to bind to gene promoter and activate gene expression (Martin et al. 2021; Chen et al. 2023). Numerous studies have demonstrated the synergistic function of HAT-TF complexes in plant development and stress responses (Dahro et al. 2022; Song et al. 2022). However, the potential role of this mechanism in regulating citric acid metabolism remains to be investigated.

This study examines the differences in citric acid content of navel oranges cultivated in Songyang county and Ganzhou city. We identified a GATA transcription factor, CitGATA7, which functions as a positive regulator of citric acid degradation. CitGATA7 plays a crucial role in the transcriptional regulation of the GS pathway associated genes, including *CitACO3*, *CitIDH1*, and *CitGS1*. Furthermore, CitGATA7 physically interacts with a histone acetyltransferase, CitHAG28, enhancing histone 3 acetylation (H3ac) levels near the transcription start site (TSS) of *CitACO3*, *CitIDH1*, and *CitGS1*, thereby promoting gene transcription. The findings reveal a novel regulatory module comprising the CitGATA7-CitHAG28 complex, which modulates citric acid catabolism by mediating expression of the GS pathway associated genes, both transcriptionally and epigenetically. This research elucidates the molecular mechanisms underlying citric acid degradation and provides insights for strategies to improve the moderate-low acidity traits in citrus breeding.

## Results

### Analysis of citric acid content and gene expression of 'SY' and 'GZ' citrus fruit

**Fig. 1:**
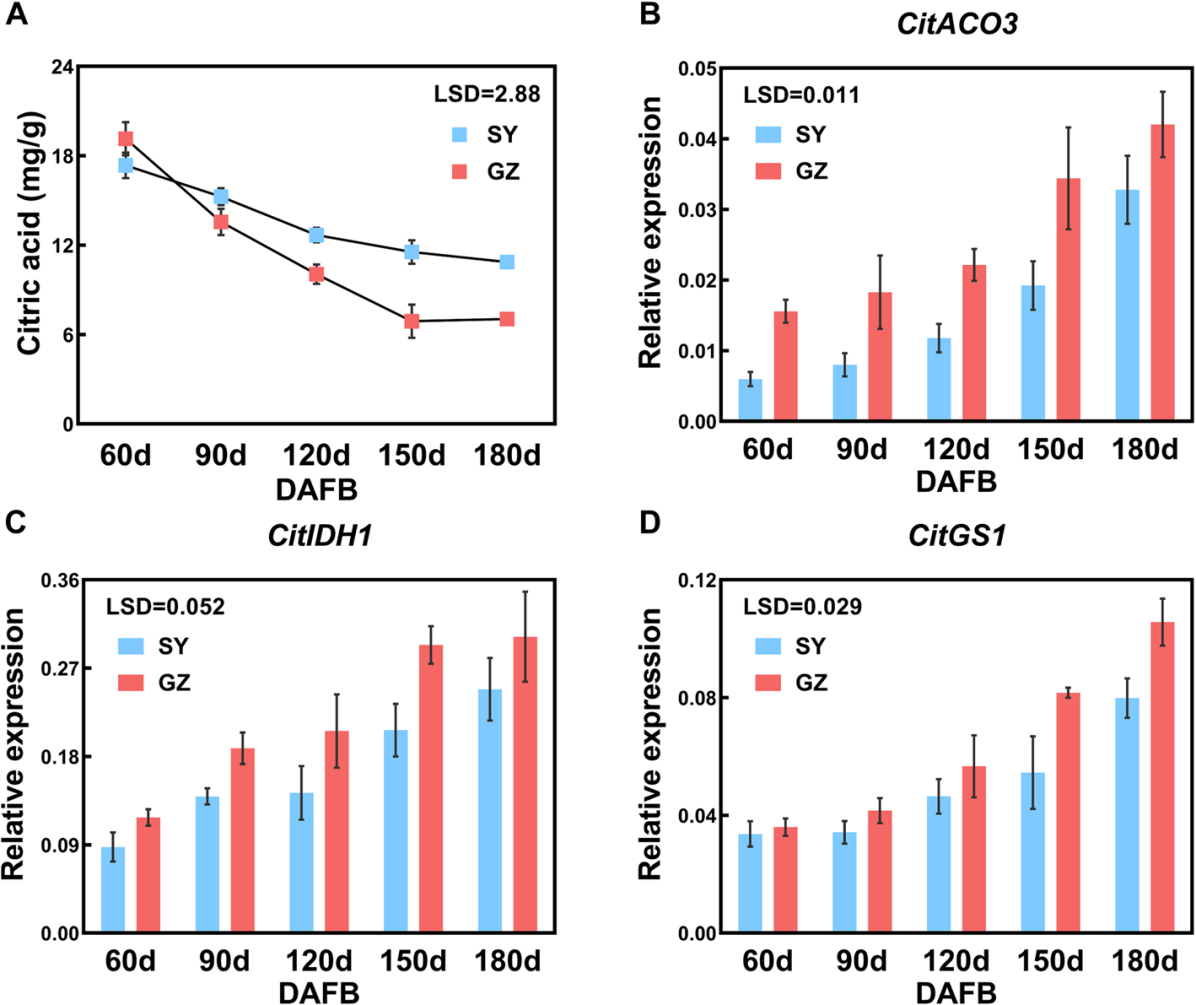
Citric acid profiles and the expression of GS pathway associated gene in 'SY' and ‘GZ’. **A** Citric acid content of 'SY' and 'GZ' fruit during fruit developmental stages. **B** Expression of *CitACO3*. **C** Expression of *CitIDH1*. **D** Expression of *CitGS1*. Error bars represent the SE (*n* = 3). LSD values were calculated at *p* = 0.05. ACO, aconitase; DAFB, days after full bloom; GS, glutamine synthetase; IDH, isocitrate dehydrogenase

The citric acid content at 5 developmental stages of 'SY' citrus fruit and 'GZ' citrus fruit was determined, ranging from 60 to 180 days after full bloom (DAFB). Both 'SY' and 'GZ' exhibited gradual decreases in citric acid content during fruit development, with 'GZ' fruit showing a more rapid decrease. Specifically, the citric acid content in 'SY' was 67% and 54% higher than in 'GZ' at 150 DAFB and 180 DAFB, respectively (Fig. 1A). Total rainfall and average relative humidity in Ganzhou city were 22.7% and 6.9% lower than in Songyang county, while the total sunshine hours and average temperature were 22.9% and 3.9% higher (Table S1). Citric acid in both 'SY' fruit and 'GZ' fruit decreased continuously from 60 DAFB, suggesting that citric acid was degraded faster than that was synthesized. Therefore, this study focused on citric acid degradation-related genes. To investigate the potential genes responsible for the citric acid difference between 'SY' and 'GZ' citrus fruit, the expression of citric acid degradation-related genes was quantified, including 3 *CitACLs* (*CitACLα1*, *CitACLα2*, and *CitACLβ1*), 3 *CitACOs* (*CitACO1*, *CitACO2*, and *CitACO3*), 3 *CitIDHs* (*CitIDH1*, *CitIDH2*, and *CitIDH3*), 2 *CitGADs* (*CitGAD4* and *CitGAD5*), and 2 *CitGSs* (*CitGS1* and *CitGS2*). The results revealed that GS pathway-associated genes, *CitACO3*, *CitIDH1*, and *CitGS1* exhibited similar expression patterns in both 'SY' and 'GZ' citrus fruit development. These genes showed lower expression in 'SY' compared to 'GZ' fruit at corresponding periods and increased gradually from 60 to 180 DAFB (Fig. 1B to D), which was significantly negatively correlated with the content of citric acid (Fig. S2A to C). However, the expression of other genes, including *CitACLs* and *CitGADs,* did not match (Fig. S1). These findings suggest that the GS pathway associated with citric acid degradation might play a crucial role in causing citric acid variation between 'SY' and 'GZ' citrus fruit.

### Identification and function analysis of CitGATA7 transcription factor

**Fig. 2:**
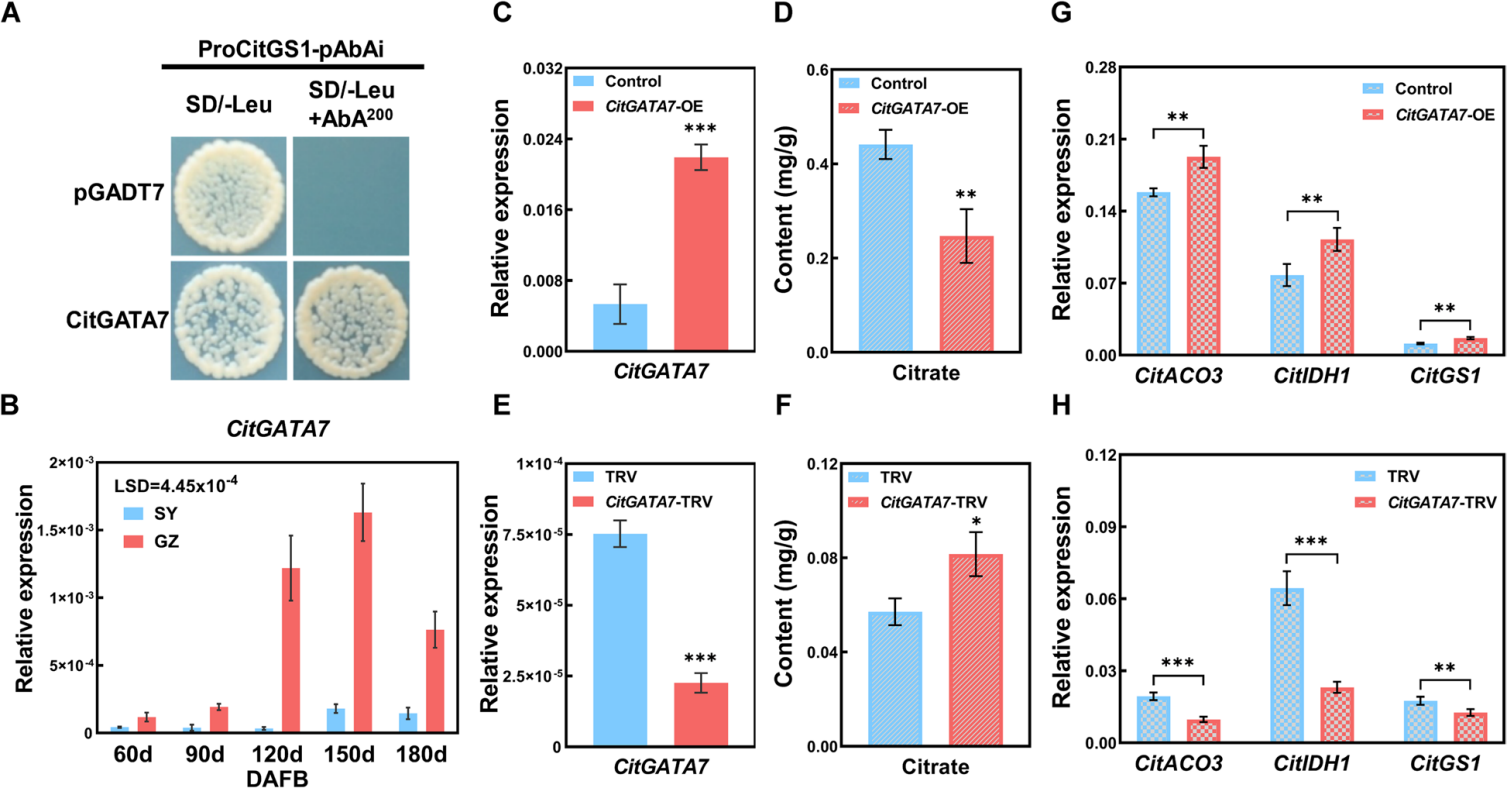
Function characterization of CitGATA7. **A** Growth of yeast cells transformed with *CitGS1* as baits and *CitGATA7* as prey on the selective medium SD/-Leu added with or without AbA (200 ng/mL). The empty pGADT7 vector was used as a negative control. **B** Expression pattern of *CitGATA7* in ‘SY’ and ‘GZ’ fruit. LSD values were calculated at *p* = 0.05. **C** Transcript level of *CitGATA7* in *CitGATA7*-OE citrus callus. **D** Citric acid content in *CitGATA7*-OE citrus callus. **E** Transcript level of *CitGATA7* in *CitGATA7*-TRV citrus seedlings. **F** Citric acid content in *CitGATA7*-TRV citrus seedlings. **G** Transcript level of *CitACO3*, *CitIDH1*, and *CitGS1* in *CitGATA7*-OE citrus callus. **H** Transcript level of *CitACO3*, *CitIDH1*, and *CitGS1* in *CitGATA7*-TRV citrus seedlings. Error bars represent the SE (*n* = 3). Asterisks indicate statistically significant differences as determined by Student's *t*-test (*p < 0.05; **p < 0.01; ***p < 0.001). AbA, aureobasidin A; ACO, aconitase; GS, glutamine synthetase; IDH, isocitrate dehydrogenase; SD, synthetic dropout

Due to the self-activation of *CitACO3* and *CitIDH1* promoters, yeast one-hybrid (Y1H) library screening was conducted using *CitGS1* as bait. Sequencing of five clones revealed CitGATA7 as the most frequent occurrence (Fig. 2A, Table S2). Subcellular localization analysis in transgenic tobacco leaves with nuclear localization signal (NLS) demonstrated that CitGATA7 is a nuclear-localized TF (Fig. S3).

To further investigate the function of CitGATA7, the expression level of *CitGATA7* was analyzed in 'SY' and 'GZ' fruit. The expression was consistently higher in 'GZ' fruit compared to 'SY' fruit, with a notable 36-fold difference at 120 DAFB (Fig. 2B). The expression pattern of *CitGATA7* exhibited a significant negative correlation with citric acid content (*r* = -0.7276, Fig. S2D), suggesting its potential role in citric acid degradation. To explore *CitGATA7* function, stable overexpression in citrus callus and virus-induced gene silencing (VIGS) in citrus seedlings were employed. Real-time fluorescent quantitative PCR (RT-qPCR) and GUS staining confirmed successful overexpression of *CitGATA7* in citrus callus (Fig. 2C, Fig. S4). The citric acid content was approximately 43% lower in the *CitGATA7*-OE callus compared to the control (Fig. 2D). Conversely, silencing *CitGATA7* in citrus seedlings significantly increased citric acid content by approximately 67% (Fig. 2E and F). These findings suggest that CitGATA7 functions as a positive regulator of citric acid degradation.

**Fig. 3:**
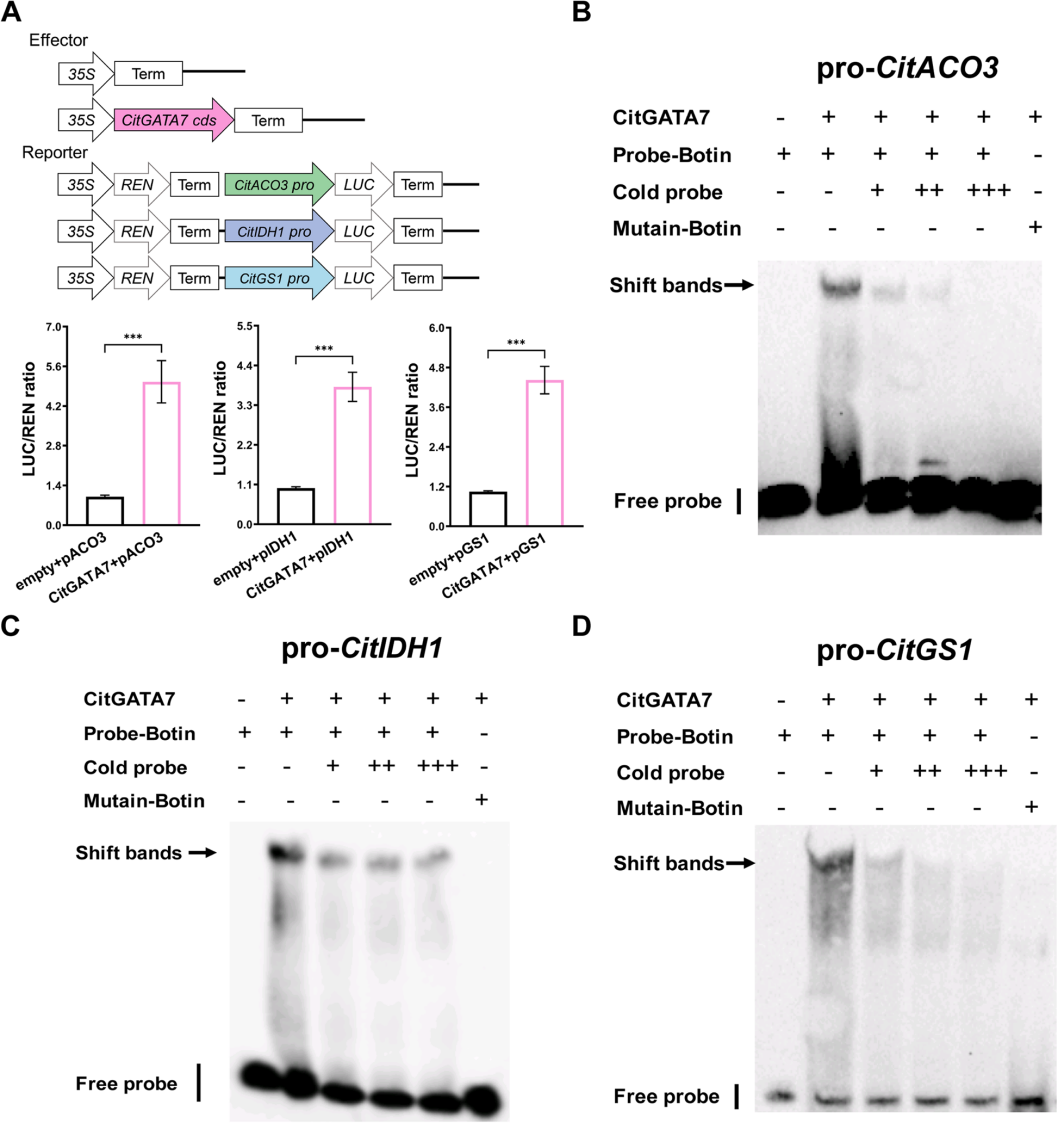
Regulatory effect of CitGATA7 on the promoter activities of the GS pathway associated gene. **A** The dual-luciferase assays evaluate CitGATA7's capacity to activate the promoter of *CitACO3*, *CitIDH1*, and *CitGS1*. The schematic map of effector and reporter indicated the *CitGATA7*-SK construct and promoter-LUC construct. The ratio of firefly LUC to REN for the empty vector (SK) plus gene promoter was set as 1. Error bars represent the SE (*n* = 4). Asterisks denote significant differences using Student's *t*-test (***p < 0.001). EMSA assay to detect CitGATA7 binding to the promoter of *CitACO3* (**B**), *CitIDH1* (**C**), and *CitGS1* (**D**). The symbol '-' and ' + ' denote the absence and presence of the component shown, respectively. ' + + ' and ' + + + ' indicate the degree of increasing amounts. ACO, aconitase; EMSA, electrophoretic mobility shift assay; GS, glutamine synthetase; IDH, isocitrate dehydrogenase; LUC, firefly luciferase; REN, Renilla luciferase

Given that *CitACO3*, *CitIDH1*, and *CitGS1* exhibited expression patterns similar to *CitGATA7*, their expression levels were examined in *CitGATA7*-OE callus and *CitGATA7*-TRV seedlings. The findings revealed that the expression of *CitACO3*, *CitIDH1*, and *CitGS1* was significantly upregulated in *CitGATA7*-OE callus (Fig. 2G), and downregulated in *CitGATA7*-TRV citrus seedlings compared to the respective controls (Fig. 2H), indicating that CitGATA7 may regulate citric acid catabolism via the GS pathway. Dual-luciferase assays demonstrated that CitGATA7 could enhance the promoter activity of *CitACO3*, *CitIDH1*, and *CitGS1* by 5.4-fold, 3.8-fold, and 4.2-fold, respectively (Fig. 3A). Electrophoresis mobility shift assays (EMSA) confirmed that CitGATA7 could physically bind to the biotin-labeled promoters of *CitACO3* and *CitGS1* in a GATCA motif-dependent manner (Fig. 3B and D), and to *CitIDH1* via a CATCA motif (Fig. 3C). The binding was attenuated by adding unlabeled WT probes in a dose-dependent manner and did not occur with mutant probes. These results suggest that CitGATA7 directly binds to the promoters and activates the transcription of *CitACO3*, *CitIDH1*, and *CitGS1*.

### CitHAG28 downregulates citric acid accumulation and interacts with CitGATA7

**Fig. 4:**
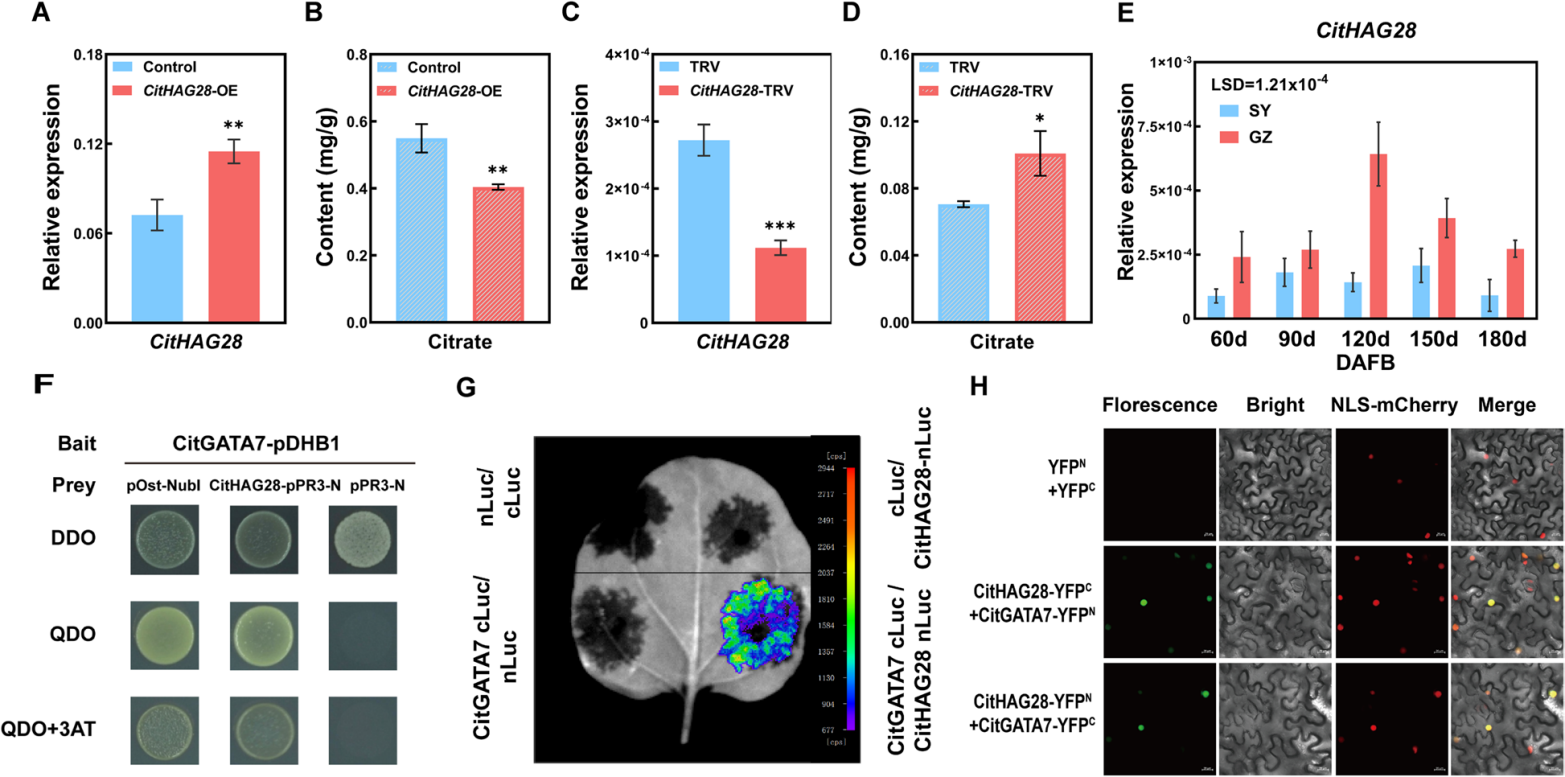
Functional analysis of CitHAG28 and its protein interaction with CitGATA7. **A** Transcript levels of *CitHAG28* in *CitHAG28*-OE citrus callus. **B** Citric acid content in *CitHAG28*-OE citrus callus. **C** Transcript levels of *CitHAG28* in *CitHAG28*-TRV citrus seedlings. **D** Citric acid content in *CitHAG28*-TRV citrus seedlings. **E** Expression pattern of *CitHAG28* in ‘SY’ and ‘GZ’ fruit. Error bars represent the SE (*n* = 3). Asterisks denote significant differences using Student's *t*-test (*p < 0.05; **p < 0.01; ***p < 0.001). LSD values were calculated at *p* = 0.05. **F** Y2H assays showing the interaction between CitGATA7 and CitHAG28. Transformants were spotted on SD/-Trp/-Leu (DDO), SD /-Trp/-Leu/-His/-Ade (QDO), or QDO with 3-AT (1 mM) media. pOst1-NubI, positive control; pPR3-N, negative control. **G** LCI assays showing the interaction between CitGATA7 and CitHAG28 in *Nicotiana benthamiana* leaves. The color bar ranges from purple to red suggesting that the luciferase activity strength is from weak to strong. **H** BIFC assay showing the interaction between CitGATA7 and CitHAG28 in transgenic *N. benthamiana* leaves. The fluorescence of GFP represents protein–protein interactions. Bar = 20 μm. 3-AT, 3-amino-1,2,4-triazole; BIFC, bimolecular fluorescence complementation; GFP, green fluorescence protein; LCI, luciferase complementation imaging; SD, synthetic dropout; Y2H, yeast two-hybrid

Our previous research indicated that the histone acetyltransferase encoding gene *CitHAG28* potentially plays a role in citric acid metabolism (Lin et al. 2024). To confirm the function of *CitHAG28* in citric acid metabolism, stable overexpression and VIGS assays were conducted. The results revealed that citric acid content in *CitHAG28*-OE citrus callus decreased by approximately 27%, while in *CitHAG28*-silenced citrus seedlings, it increased by approximately 43% compared to the control (Fig. 4A to D). These findings demonstrate that CitHAG28 negatively regulates citric acid accumulation.

The expression of *CitHAG28* in 'SY' and 'GZ' citrus fruit was further quantified. *CitHAG28* expression in 'GZ' increased rapidly from 90 to 120 DAFB, and subsequently decreased, while no significant changes were observed in 'SY' (Fig. 4E). The higher expression level of *CitHAG28* in 'GZ' fruit compared to 'SY' fruit was similar to that of *CitGATA7* (Figs. 2B and 4E), suggesting a potential synergistic effect between CitHAG28 and CitGATA7. To test this hypothesis, yeast two-hybrid (Y2H), bimolecular fluorescence complementation (BiFC), and luciferase complementation imaging (LCI) assays were conducted. The results demonstrated that CitGATA7 interacted with CitHAG28, which occurred in the nucleus (Fig. 4F to H).

### CitHAG28 enhances the histone acetylation levels near TSS of *CitACO3*, *CitIDH1*, and *CitGS1*

**Fig. 5:**
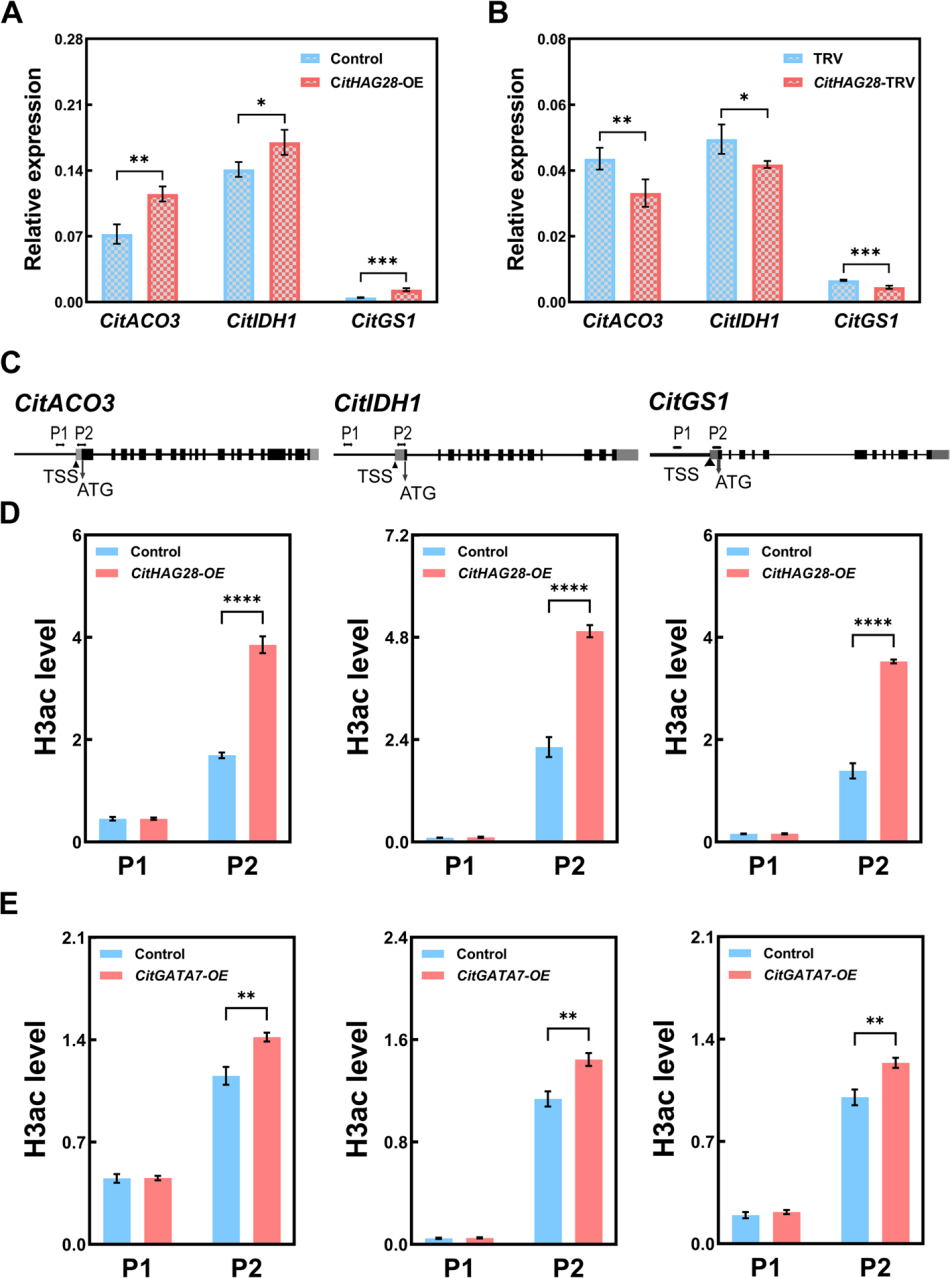
Regulatory effects of CitHAG28 on the GS pathway associated gene. **A** Expression patterns of *CitACO3*, *CitIDH1*, and *CitGS1* in *CitHAG28*-OE citrus callus. **B** Expression patterns of *CitACO3*, *CitIDH1*, and *CitGS1* in *CitHAG28*-TRV citrus seedlings. **C** The schematic map of primer design positions for ChIP-qPCR assay. **D** The levels of H3ac around the promoter and near the TSS of *CitACO3*, *CitIDH1*, and *CitGS1* in *CitHAG28*-OE citrus callus. **E** The levels of H3ac around the promoter and near the TSS of *CitACO3*, *CitIDH1*, and *CitGS1* in *CitGATA7*-OE citrus callus. Error bars represent the SE (*n* = 3). Asterisks denote significant differences using Student's *t*-test (*p < 0.05; **p < 0.01; ***p < 0.001). ACO, aconitase; ChIP, chromatin immuno-precipitation; GS, glutamine synthetase; H3ac, Histone 3 acetylation; IDH, isocitrate dehydrogenase; TSS, transcription start site

Given the interaction between CitHAG28 and CitGATA7, the study examined the expression of CitGATA7's target genes, *CitACO3*, *CitIDH1*, and *CitGS1*, in both *CitHAG28*-OE citrus callus and *CitHAG28*-TRV citrus seedlings. The results revealed a significant upregulation of C*itACO3*, *CitIDH1*, and *CitGS1* expression in *CitHAG28*-OE citrus callus compared to the control (Fig. 5A). Conversely, a marked decrease in expression was observed in *CitHAG28*-TRV citrus seedlings (Fig. 5B). These findings indicate that *CitACO3*, *CitIDH1*, and *CitGS1* are likely shared target genes of both CitGATA7 and CitHAG28.

To investigate whether CitHAG28 enhances histone acetylation levels near the TSS of *CitACO3*, *CitIDH1*, and *CitGS1*, the study analyzed the occupancy of H3ac and histone 4 acetylation (H4ac) in the regions near CitGATA7 binding site as well as the TSS (Fig. 5C). The findings revealed that the H3ac levels near the TSS of *CitACO3*, *CitIDH1*, and *CitGS1* were significantly elevated in *CitHAG28*-OE compared to the control, while no significant difference was observed near CitGATA7 binding site (Fig. 5D). Furthermore, the H4ac level near the TSS of target genes showed no significant change (Fig. S5). These results indicate that CitHAG28 upregulates the transcript levels of *CitACO3*, *CitIDH1*, and *CitGS1* by specifically mediating the H3ac level near the TSS region.

### CitGATA7 mediates histone acetylation levels of target genes via CitHAG28

**Fig. 6:**
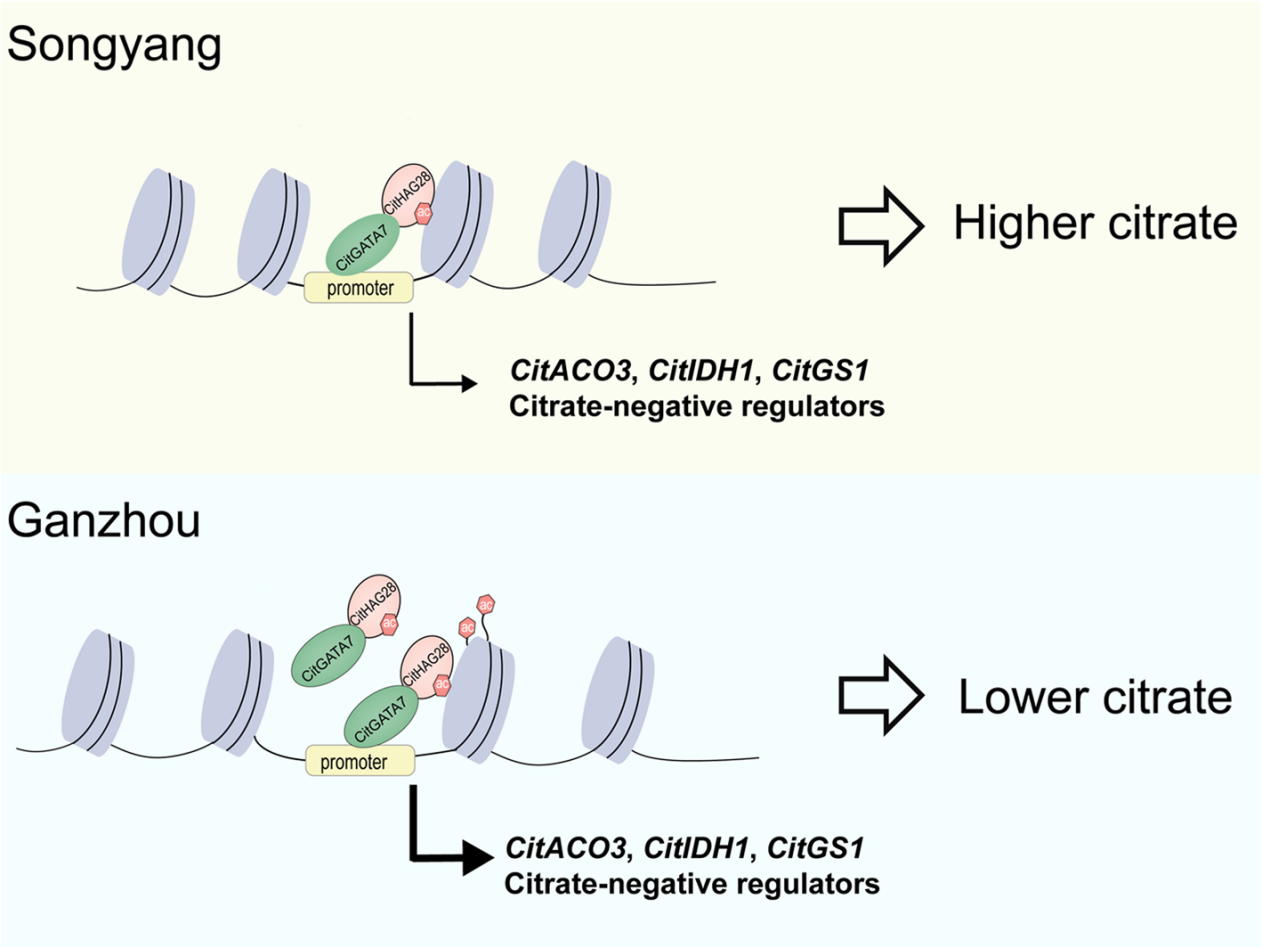
A proposed model of CitGATA7-CitHAG28 protein complex in the regulation of citrate degradation via GS pathway in citrus fruit. In the "Newhall" navel orange cultivated in Songyang county, the expression of *CitGATA7* and *CitHAG28* was suppressed, leading to reduced expression of *CitACO3*, *CitIDH1*, and *CitGS1*, resulting in elevated citric acid levels. Conversely, in the "Newhall" navel orange grown in Ganzhou city, *CitGATA7* and *CitHAG28* were highly expressed, forming a protein complex that loosened the chromatin structure and activated the transcription of *CitACO3*, *CitIDH1*, and *CitGS1*, leading to lower citric acid levels. ACO, aconitase; GS, glutamine synthetase; IDH, isocitrate dehydrogenase

The interaction between CitGATA7 and CitHAG28 suggested that CitGATA7 may influence the histone acetylation level near the TSS of *CitACO3*, *CitIDH1*, and *CitGS1*. To test this hypothesis, the H3ac and H4ac levels were examined in the control and *CitGATA7*-OE citrus callus. The results indicated that the H3ac levels near the TSS of C*itACO3*, *CitIDH1*, and *CitGS1* were significantly higher in *CitGATA7*-OE callus compared to the control, while no significant difference was observed in the H3ac level around the promoter region (Fig. 5E). Furthermore, no significant difference was found in H4ac levels (Fig. S5). These findings confirm that CitGATA7 could recruit CitHAG28 to modify the H3ac levels near the TSS of *CitACO3*, *CitIDH1*, and *CitGS1*, thereby enhancing gene expression (Fig. 6).

## Discussion

### Environment affects citric acid metabolism during citrus fruit development

Citric acid accumulation patterns vary among citrus fruit varieties during development from fruit expansion to ripening. In most varieties, citric acid rapidly accumulates during early fruit expansion, reaches a peak, and then decreases (Lin et al. 2015; Lin et al. 2016). Lemon, however, maintains high citric acid levels after peaking, with only a slight decrease during later ripening stages (Sadka et al. 2000a). This study observed that in "Newhall" navel orange, citric acid content peaked at 60 DAFB, decreased from 60 to 150 DAFB, and then remained relatively stable from 150 to 180 DAFB in 'SY' fruit, with a slight increase in 'GZ' fruit (Fig. 1A), similar to patterns in "Satsuma" and "Ponkan". Contrastingly, Zhou et al. (2018) reported that citric acid in "Newhall" oranges grown in Wuhan city, Hubei Province, peaked around 90 DAFB, suggesting that environmental factors may influence citrus acid metabolism patterns (Hussain et al. 2017; Zhou et al. 2018; Zhang et al. 2024).

Plants acclimate and adapt to changing environments through epigenetic inheritance, transmitting this memory to their offspring (Gallusci et al. 2023). Environmental factors influence epigenetic modifications, which in turn regulate fruit quality (Zhu et al. 2023; Li et al. 2020b). Citric acid metabolism is also subject to environmental regulation (Zheng et al. 2019; Jiang et al. 2022). In this study, the temperature difference between Songyang county and Ganzhou city is minimal at 0.81℃. However, Ganzhou city experienced 22.7% less total rainfall and 22.9% more total sunshine hours compared to Songyang county (Table S1). These findings suggest that temperature may not be the primary factor contributing to the differences in citric acid content between 'SY' and 'GZ' fruits; instead, sun exposure duration or water availability may be more influential. Notably, previous research has shown that histone acetylation levels increase under drought stress in *Populus* and high irradiation in maize, inducing stress tolerance (Li et al. 2019; Casati et al. 2008). It is important to note that the effects of environmental factors on epigenetic regulation and fruit development are dynamic. Further research is necessary to conclusively identify the specific environmental factor(s) determining citric acid accumulation in citrus fruits.

### GS pathway is important for the degradation of citric acid in 'SY' and 'GZ' citrus fruit

Citric acid usually rapidly accumulates in the early stage of citrus fruit, and then degrades (Hussain et al. 2017). The reduction in citric acid content was correlated with increased expression of genes associated with citric acid degradation, including the ACL pathway, the GABA shunt pathway, and the GS pathway (Sadka et al. 2000b; Chen et al. 2013; Zhou et al. 2018). Gene expression analysis of citric acid degradation-related genes in 'SY' and 'GZ' citrus fruit revealed that the expression of *CitACLs* in the ACL pathway and *CitGADs* in the GABA shunt did not correlate significantly with citric acid content in either cultivar (Fig. S1). However, the expression levels of *CitACO3*, *CitIDH1*, and *CitGS1* were consistently higher in 'GZ' fruit compared to 'SY' fruit, particularly during the period of citric acid decline from 90 to 150 DAFB (Fig. 1B to D). Consequently, the elevated expression of *CitACO3*, *CitIDH1*, and *CitGS1* in the GS pathway appears to be responsible for the accelerated degradation of citric acid in 'GZ' fruit. This finding aligns with Chen et al. (2013) suggesting that the GS pathway may play a crucial role in citric acid degradation across different environmental conditions.

### CitGATA7 is a key transcriptional regulator of citric acid degradation

Citric acid metabolism is influenced by various metabolic enzymes, acid transporters, proton pumps, and TFs. Research on the transcriptional regulation of citric acid metabolism in citrus has primarily focused on transportation and degradation. Studies have shown that CitAN1 (a bHLH transcription factor), CitPH4 (an R2R3 MYB transcription factor), and CitTRL (an R3-MYB transcription factor) affect citric acid accumulation by regulating the expression of *CitPH5* (a vacuolar P-ATPases) (Butelli et al. 2019; He et al. 2022; Huang et al. 2023). Additionally, CitERF6 and CitMYB52-CitbHLH2 transactivate the promoters of *CitACL1* and *CitALMT*, respectively, promoting citric acid degradation (Li et al. 2020a; Liu et al. 2022). The GS pathway associated gene, *CitACO3*, is synergistically regulated by CitNAC62-CitWRKY1 and also by CitHsfA7 (Li et al. 2017b, 2022). However, previous transcriptional regulation studies have mainly focused on single regulation, with limited research on master TF regulating the entire pathway. In this study, CitGATA7 was found to simultaneously transactivate the promoter activity of the GS pathway associated genes *CitACO3*, *CitIDH1*, and *CitGS1* (Fig. 3), potentially due to the ability of GATA transcription factors to bind multiple elements (Chen et al. 2012; Shen et al. 2021). Phenotypic analyses revealed that when *CitGATA7* was overexpressed at only twofold higher than the control, citric acid levels in citrus callus were reduced by approximately 43%. Conversely, citric acid levels increased by about 46% in *CitGATA7* gene-silenced seedlings compared to the control (Fig. 2C to F), indicating that CitGATA7 is a potent positive regulator of citric acid degradation through simultaneous regulation of multiple genes in the GS pathway. These findings not only present a core TF of the GS pathway but also enhance the existing regulatory network of citric acid metabolism TFs.

Citric acid plays a crucial role in plants' ability to manage nutrient deficiencies and cope with abiotic and biotic stresses. GATA transcription factors, characterized by a distinctive zinc finger DNA binding domain, are integral to plant resistance against abiotic adversity (Gupta et al. 2017; Bastakis et al. 2018; Schroder et al. 2023). Recent research has demonstrated that PpGATA12 responds to the 24-epibrassinolide (EBR) signal by enhancing the transcription of *PpSS* (a sucrose synthase) and *PpNI * (a neutral invertase), thereby mitigating cold damage during storage through the regulation of sucrose metabolism in peach (Hu et al. 2023). This finding further supports the conserved resistance function of GATA. Our study, which elucidates CitGATA7's role in citric acid regulation, may expand the understanding of the diverse functions of GATA transcription factors in plants. Further investigation is needed to determine whether CitGATA7 is involved in other organic acid metabolic pathways and to explore its potential role in stress resistance.

### CitHAG28 is a key epigenetic regulator of citric acid degradation

Our previous research demonstrated that citric acid content in citrus callus increased following treatment with the HAT inhibitor γ-butyrolactone (MB-3), suggesting the potential involvement of *CitHAG28* in citric acid metabolism (Lin et al. 2024). The present study reveals distinct expression patterns of *CitHAG28* in 'SY' and 'GZ' citrus fruits (Fig. 4E). Furthermore, we established that CitHAG28 positively regulates the expression of *CitACO3*, *CitIDH1*, and *CitGS1* by enhancing H3ac levels and promoting citric acid degradation (Fig. 4B and D 5A and B). These findings indicate that histone acetylation mediates the differential citric acid content observed in 'SY' and 'GZ' fruits.

DNA wraps around histone octamers to form nucleosomes, and the histone acetylation level can be analyzed by quantifying the precipitated chromatin DNA (Chen et al. 2022). Histone acetylation is predominantly enriched around the promoter region, particularly near the transcription start site (Kumar et al. 2018; Kim et al. 2020; Tang et al. 2022; Zhu et al. 2023). Furthermore, the + 1 nucleosome (the first downstream nucleosome of the TSS) can recruit transcriptional regulators through histone modifications to enhance transcription (Chen et al. 2022). To investigate the function of CitHAG28 histone acetylation, the H3ac and H4ac levels near the TSS of *CitACO3*, *CitIDH1*, and *CitGS1* were quantified. The results revealed significantly higher H3ac levels near the TSS of *CitACO3*, *CitIDH1*, and *CitGS1* in *CitHAG28*-OE citrus callus compared to the control (Fig. 5D), while their H4ac levels remained unchanged (Fig. S5A). These findings suggest that CitHAG28 accelerates citric acid degradation by specifically increasing the H3ac level to promote the expression of *CitACO3*, *CitIDH1*, and *CitGS1*. This is consistent with the conclusion that high histone acetylation is evidence of HAT-activated gene expression (Lin et al. 2022; Song et al. 2022; Zhao et al. 2023).

### CitGATA7-CitHAG28 protein complex co-regulate citric acid degradation by modulating GS pathway

Gene expression is co-regulated by TFs and epigenetic modification regulators (Xiong et al. 2024). TFs regulate gene expression by binding to specific DNA sequences, while epigenetic regulators exert a synergistic effect by modulating chromatin structure to facilitate or inhibit TF binding (Allis et al. 2016; Chen et al. 2022). Research on the synergistic regulation of fruit quality formation and ripening by histone acetylation and transcriptional regulation has primarily focused on HDAC-TF complexes. Recent studies have demonstrated that SlERF.F12-SlTPL2-SlHDA1/3 negatively modulates tomato fruit ripening (Deng et al. 2022), MdERF4-MdTPL4-MdHDA4 functions as a repressor complex reducing *MdACS3a* expression to regulate apple fruit ripening (Hu et al. 2022), and VvERF4 and VvHDAC19 negatively regulate anthocyanin biosynthesis and accumulation in grapes (Jia et al. 2023). However, studies on the synergistic effects of HAT with TF have mainly focused on model plants such as *Arabidopsis* and rice, with limited research involving fruit quality (Li et al. 2020b; Song et al. 2022). Previously, GATA transcription factors were reported to co-regulate biological processes with histone demethylase, including flowering time and seed dormancy regulation (Yang et al. 2022; Wei et al. 2023). In this study, we found that CitGATA7 forms a protein complex with CitHAG28 in the nucleus (Fig. 4H). Further results indicated that the H3ac levels near the TSS of *CitACO3*, *CitIDH1*, and *CitGS1* were significantly higher in *CitGATA7*-OE citrus callus compared to the control, albeit to a lesser extent than in *CitHAG28*-OE citrus callus (Fig. 5E), whereas there was no significant difference in H4ac level (Fig. S5B). These findings suggest that CitGATA7 recruits CitHAG28 for H3ac, enhancing the activation effects on the promoters of GS pathway-associated genes *CitACO3*, *CitIDH1*, and *CitGS1*, thereby accelerating citric acid degradation in citrus. This study not only deepens the understanding of the link between histone acetylation and transcriptional regulation but also complements the known functions of HAT in fruit quality.

## Materials and Methods

### Plant materials

'SY' citrus fruit (*C. sinensis* Osbeck cv. Newhall) and 'GZ' citrus fruit (*C. sinensis* Osbeck cv. Newhall) were sourced from Songyang county, Zhejiang province and Ganzhou city, Jiangxi province, respectively. Fruits of uniform appearance and size were harvested at 60, 90, 120, 150, and 180 DAFB, with three biological replicates. The fruit flesh was flash-frozen in liquid nitrogen and stored at -80 °C for further analysis.

Embryonic citrus callus (*C. sinensis* cv. Valencia) was cultivated in a medium containing 4.43 g/L MT basal medium, 40 g/L sucrose, 8 g/L agar, and 5 mg/L L-ascorbic acid, with pH adjusted to 5.8. The culture was maintained at 25 °C under a 16 h/8 h light/dark cycle. Citrus seeds (*C. sinensis* cv. Valencia) were germinated in medium and subsequently grown in soil for 1.5 months. The leaves from these seedlings were harvested and stored at -80 °C for future analyses.

### Citric acid quantification

Citric acid content was determined according to the method described by Lin et al. (2024). A 0.1 g sample was combined with 1.4 mL of chromatographic methanol at 70 °C, mixed for 15 min, and centrifuged at 4℃, 11000 g for 10 min. The resulting upper phase was mixed with 0.75 mL trichloromethane and 1.5 mL purified water, then centrifuged at 2200 g for 10 min. A 100 μL aliquot of the supernatant was transferred to a 1.5 mL tube containing 20 μL of ribitol (2 mg/mL) for vacuum drying. The dried pellet was subsequently dissolved in 60 μL of 20 mg/mL pyridine methoxyamine hydrochloride and incubated at 950 rpm at 37℃ for 1.5 h. Finally, the solution was mixed with 40 μL of bis (trimethylsilyl) trifluoroacetamide (1% trimethylchlorosilane) for 0.5 h under identical conditions. Quantification of the sample's citric acid content was performed using a Gas Chromatograph/Mass Selective Detector (7890N-5975C, Agilent, USA).

### RNA extraction and RT-qPCR

RNA extraction was conducted following a modified version of the protocol described by Chang et al. (1993). cDNA synthesis was performed using the HiScript III 1st strand cDNA synthesis kit (Vazyme, Nanjing, China). RT-qPCR assays were performed on a Bio-Rad CFX96 instrument (Bio-Rad, Marnes la Coquette, France) utilizing the AceQ qPCR SYBR green master mix (Vazyme, Nanjing, China) as detailed in Ren et al. (2023). Gene transcript levels were quantified using the citrus *β-actin* gene (Ciclev10025866m.g) and calculated by 2^-ΔCt^ (Li et al. 2017b). The primers used for RT-qPCR are listed in Table S3.

### Screening of the cDNA library

The fragmented promoter of *CitGS1* was amplified and inserted into the pAbAi vector to serve as bait. The recombinant plasmid, following linearization by *Bst*BI digestion, was integrated into the Y1H gold yeast strain. A Y1H cDNA library was constructed using total RNA extracted from navel orange. The target protein was utilized as bait in the Matchmaker Gold Yeast One-Hybrid Library Screening System (Clontech, USA).

### Subcellular localization analysis

The coding sequences (CDS) of *CitGATA7* and *CitHAG28* without the stop codon were individually fused to the pCAMBIA1300-GFP vector (Chalfie et al. 1994). The constructs and empty vector (35S::GFP) were transformed into *A. tumefaciens* (GV3101), and subsequently co-infiltrated into the leaves of transgenic *Nicotiana benthamiana* expressing *H2B-RFP* as a nucleus-located mCherry marker (Li et al. 2017a). The GFP fluorescence signal was visualized using a Zeiss LSM710NLO confocal laser scanning microscope, with parameters as described by Fang et al. (2023). The primers used for vector construction are listed in Table S4.

### Genetic transformation of citrus callus

The complete CDS of *CitGATA7* and *CitHAG28* were inserted into the modified pCAMBIA-1301 vector (Khanna and Raina 1999). The *GUS* reporter gene with CaMV 35S promoter in the vector served as the positive control. The constructs were independently introduced into citrus callus via *A. tumefaciens* (EHA105) mediated transformation, following a previously described method with slight modifications (Li et al. 2002). The transformed callus was co-cultured on solid MT medium in darkness for 3 days, then transferred to solid MT selective medium containing 50 mg/L hygromycin and 400 mg/L cefotaxime at 25 °C in darkness, with monthly subculturing. The transgenic callus was confirmed through RT-qPCR and GUS staining kit (Real-Times Biotechnology, China).

#### VIGS

The VIGS assay was conducted based on the protocol described by Liu et al. (2002), with some modification. A specific gene fragment was inserted into the tobacco rattle virus-based vector 2, which was subsequently introduced into *A. tumefaciens* (EHA105). Bacterial suspensions were cultivated in Luria–Bertani broth medium and adjusted to an OD_600_ of 1.0 using infiltration buffer (10 mM MES, 10 mM MgCl_2_, 150 mM acetosyringone). For VIGS, bacterial suspensions containing TRV1 and the TRV2 construct at a 1:1 volume ratio were incubated for 3 h in darkness, with a mixture of TRV1 and TRV2 serving as the control. The bacterial mixture was vacuum infiltrated into germinated seedlings, which were then grown in darkness for 3 days before being transferred to a growth chamber for 30 days. RT-qPCR was employed to detect the transformed seedlings.

### Dual-luciferase assay

The dual-luciferase assay was conducted in accordance with a previous study (Qian et al. 2022). The full-length CDS of *CitGATA7* was inserted into the pGreenII 0029 62-SK vector, while the promoters of *CitACO3*, *CitIDH1*, and *CitGS1* were inserted into the pGreenII 0800-LUC vector (Hellens et al. 2005). All constructs were transformed into *A. tumefaciens* (GV3101) harboring the pSoup helper plasmid. For injections, the cultures were adjusted to an OD_600_ of 0.75 with infiltration buffer as described above, and a mixed culture (v/v, 10:1) of TF and each promoter was infiltrated into *N. benthamiana* leaves using a needle syringe. After 3 days, the firefly luciferase and renilla luciferase activities were measured using the Dual-Luciferase Reporter Assay System (Promega, America) on the GloMax 96 Microplate Luminometer (Promega, America). Each experiment was performed in quadruplicate.

### EMSA assay

The complete CDS of *CitGATA7* was inserted into the pET-32a vector containing a His tag. Protein expression and purification were performed as previously described (Ma et al. 2023). Oligonucleotides were synthesized and labeled with 3′-biotin ends. EMSA was conducted following the manufacturer's instructions for the LightShift Chemiluminescent EMSA kit (Thermo, USA). The oligonucleotide sequences are provided in Table S5.

### Y2H assay

The Dual Hunter system (Dual System Biotechnology, Switzerland) was employed to examine protein–protein interactions in yeast. The full-length CDS of *CitGATA7* or *CitHAG28* was inserted into the pDHB1 vector as bait, while the full-length *CitHAG28* or *CitGATA7* was inserted into the pPR3N vector as prey. Various combinations of the constructs were transformed into yeast strain NMY51. Colonies were cultured in liquid medium, diluted to OD_600_ = 0.002, and plated on selective media: (i) SD media without tryptophan and leucine (DDO); (ii) SD media without tryptophan, leucine, His, and Ade (QDO); (iii) QDO medium supplemented with 1 mM 3-amino-1,2,4-triazole (3-AT). Combinations of empty pPR3-N vector and pDHB1 vector containing target genes were used to assess autoactivation. Combinations of pOst1-Nub1 and pDHB1 vector containing target genes served as positive controls. Yeast cells were incubated at 30 °C for 3 days.

### BiFC assay

The CDS sequence of CitGATA7 and CitHAG28 were inserted into the C-terminal and N-terminal regions of the GFP vector (Lv et al. 2014) and subsequently transformed into *A. tumefaciens* (GV3101). The resulting fusion proteins, or one of them with empty vectors, were co-infiltrated into the leaves of transgenic *N. benthamiana* which expressed *H2B-RFP* as nucleus-located mCherry marker (Li et al. 2017a). After 48 h, GFP fluorescence signals were captured using a Zeiss LSM710NLO confocal laser scanning microscope, displaying adjusted yellow fluorescence. The parameter was according to Fang et al. (2023).

### LCI assay

The CDS of *CitGATA7* and *CitHAG28* genes were inserted into the pCAMBIA1300-nLUC vector and pCAMBIA1300-cLUC vector (Chen et al. 2008) before being transformed into *A. tumefaciens* (GV3101). The resulting fusion proteins, or one of them with empty vectors, were co-infiltrated into *N. benthamiana* leaves. After 48 h, 0.5 mM d-luciferin was applied to the infiltrated leaves, and LUC fluorescence was detected using an in vivo imaging system (LB985 NightSHADE, Germany).

### ChIP assay

A 3 g powder sample was underwent vacuum cross-linking in 1% (v/v) formaldehyde for 30 s, followed by nuclei extraction and chromatin sonication. The anti-H3ac antibody (ABclonal, China) and IgG antibody (ABclonal, China) were incubated with pierce protein A/G magnetic beads (Thermo, USA) at 4 °C for 2 h. Subsequently, the sonicated chromatin was incubated overnight. The immunoprecipitated DNA fragments were purified, and the enriched DNA fragments were utilized for RT-qPCR analysis. Five percent of the unimmunized sonicated chromatin served as input. The relevant primer sequences are presented in Table S6.

### Statistical analysis

Data analysis was conducted using Microsoft Excel and GraphPad Prism 9.0. Two-tailed tests were employed for correlation analyses, and the Pearson correlation coefficient was calculated. Statistical significance was assessed using Student's *t*-test and Least Significant Difference (LSD). Graphs and figures were created using GraphPad Prism 9.0 and Adobe Illustrator 2023, respectively.

## Supplementary Information

Supplementary Material 1: Supplementary Fig. S1 Expression patterns of 11 citric acid degradation-related genes in 'SY' and 'GZ' fruits during developmental stages. Error bars represent the standard error (*n* = 3). LSD values were calculated at* p* = 0.05. Supplementary Fig. S2 Correlation analysis between citric acid contentand gene expressions. *CitACO3* (A), *CitIDH1* (B),*CitGS1* (C), and *CitGATA7* (D)*.*Statistical significance was determined by two-tailed test (*p <0.05,**p <0.01, ***p <0.001). Supplementary Fig. S3 Subcellular localization analysis of CitGATA7 and CitHAG28 in transgenic *Nicotiana benthamiana*leaves. 35s-eGFP serves as a positive control. Bar = 20 μm. Supplementary Fig. S4 GUS staining analysis in citrus callus of the control, *CitGATA7*-OE and *CitHAG28*-OE*.* The blue coloration indicates positive citrus callus successfully transformed with the pCAMBIA1301 vector. Supplementary Fig. S5 The level of H4ac around the promoter (P1) and near the TSS (P2) of *CitACO3*, *CitIDH1*, and *CitGS1*. (A) *CitHAG28*-OE citrus callus; (B) *CitGATA7*-OE citrus callus. Error bars indicate the standard error (SE) (*n* = 3).

Supplementary Material 2: Supplementary Table S1 Habitat conditions of two navel orange orchards. Supplementary Table S2 Y1H library screening of *CitGS1 *promoter. Supplementary Table S3 Primers used for RT-qPCR. Supplementary Table S4 Primers used for vector construction. Supplementary Table S5 Primers used for EMSA assay. Supplementary Table S6 Primers used for ChIP-qPCR.

## Data Availability

Not applicable.

## Abbreviations

3-AT: 3-Amino-1,2,4-triazole
35S: Cauliflower mosaic virus promoter
AbA: Aureobasidin A
ACO: Aconitase
BiFC: Bimolecular fluorescence complementation
CDS: Coding sequences
DAFB: Days after full blossom
EMSA: Electrophoresis mobility shift assays
GFP: Green fluorescent protein
GS: Glutamine synthetase
H3ac: Histone 3 acetylation
H4ac: Histone 4 acetylation
HAT: Histone acetyltransferases
HDAC: Histone deacetylases
IDH: Isocitrate dehydrogenase
LCI: Luciferase complementation imaging
NLS: Nuclear localization signal
RT-qPCR: Reverse transcription-quantitative polymerase chain reaction
TF: Transcription factor
TSS: The transcription start site
VIGS: Virus-induced gene silencing
Y1H: Yeast one-hybrid
Y2H: Yeast two-hybrid
