# Supplementary material for: CitGATA7 interact with histone acetyltransferase CitHAG28 to promote citric acid degradation by regulating the glutamine synthetase pathway in citrus
Source: Mol Hortic. 2025 Feb 1;5:8. doi: 10.1186/s43897-024-00126-y (PMC11786515; doi:10.1186/s43897-024-00126-y)
Supplement: Supplementary file 1 — Supplementary Material 1: Supplementary Fig. S1 Expression patterns of 11 citric acid degradation-related genes in 'SY' and 'GZ' fruits during developmental stages. Error bars represent the standard error (n = 3). LSD values were calculated at p = 0.05. Supplementary Fig. S2 Correlation analysis between citric acid contentand gene expressions. CitACO3 (A), CitIDH1 (B),CitGS1 (C), and CitGATA7 (D).Statistical significance was determined by two-tailed test (*p <0.05,**p <0.01, ***p <0.001). Supplementary Fig. S3 Subcellular localization analysis of CitGATA7 and CitHAG28 in transgenic Nicotiana benthamianaleaves. 35s-eGFP serves as a positive control. Bar = 20 μm. Supplementary Fig. S4 GUS staining analysis in citrus callus of the control, CitGATA7-OE and CitHAG28-OE. The blue coloration indicates positive citrus callus successfully transformed with the pCAMBIA1301 vector. Supplementary Fig. S5 The level of H4ac around the promoter (P1) and near the TSS (P2) of CitACO3, CitIDH1, and CitGS1. (A) CitHAG28-OE citrus callus; (B) CitGATA7-OE citrus callus. Error bars indicate the standard error (SE) (n = 3). [file 43897_2024_126_MOESM1_ESM.docx]

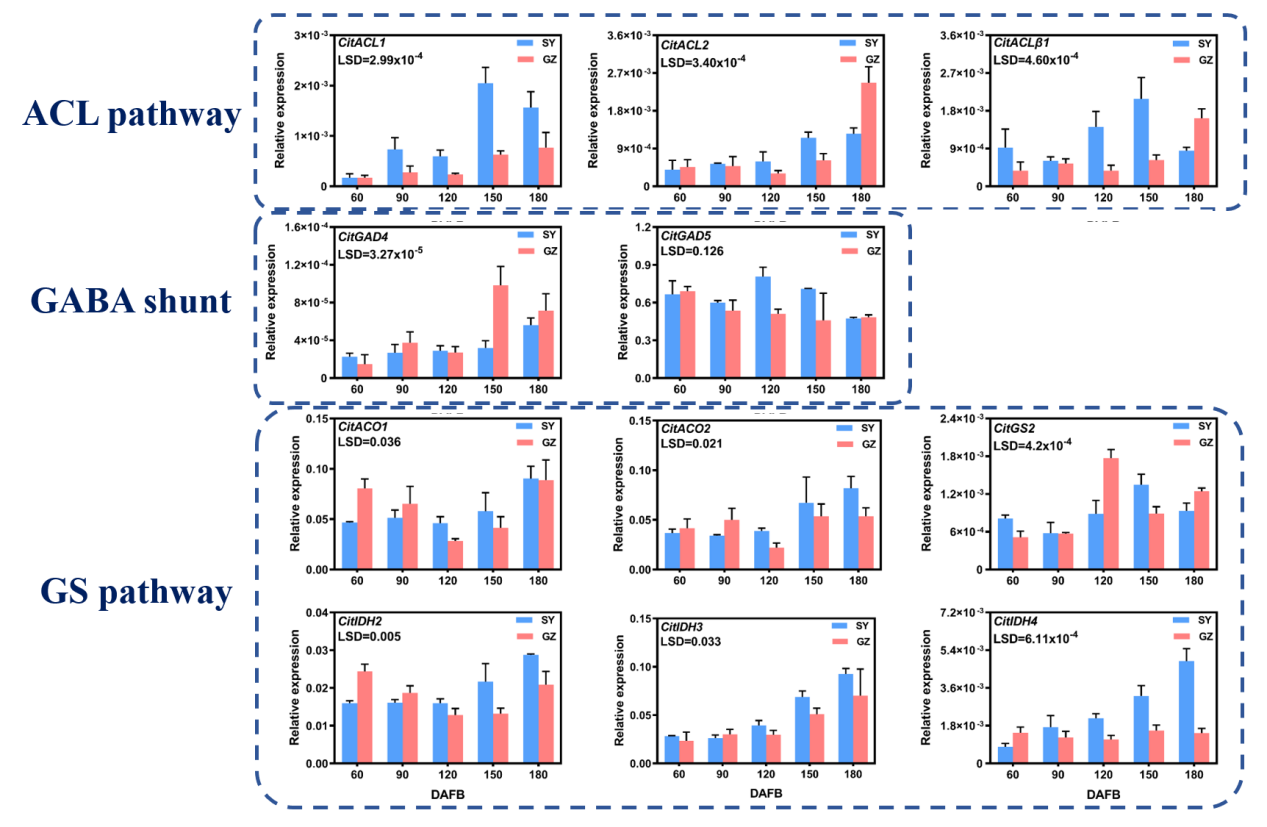


**Fig. S1** Expression patterns of 10 citric acid degradation-related genes in 'SY' and 'GZ' fruits during developmental stages. Error bars represent the standard error (*n* = 3). LSD values were calculated at *p* = 0.05.


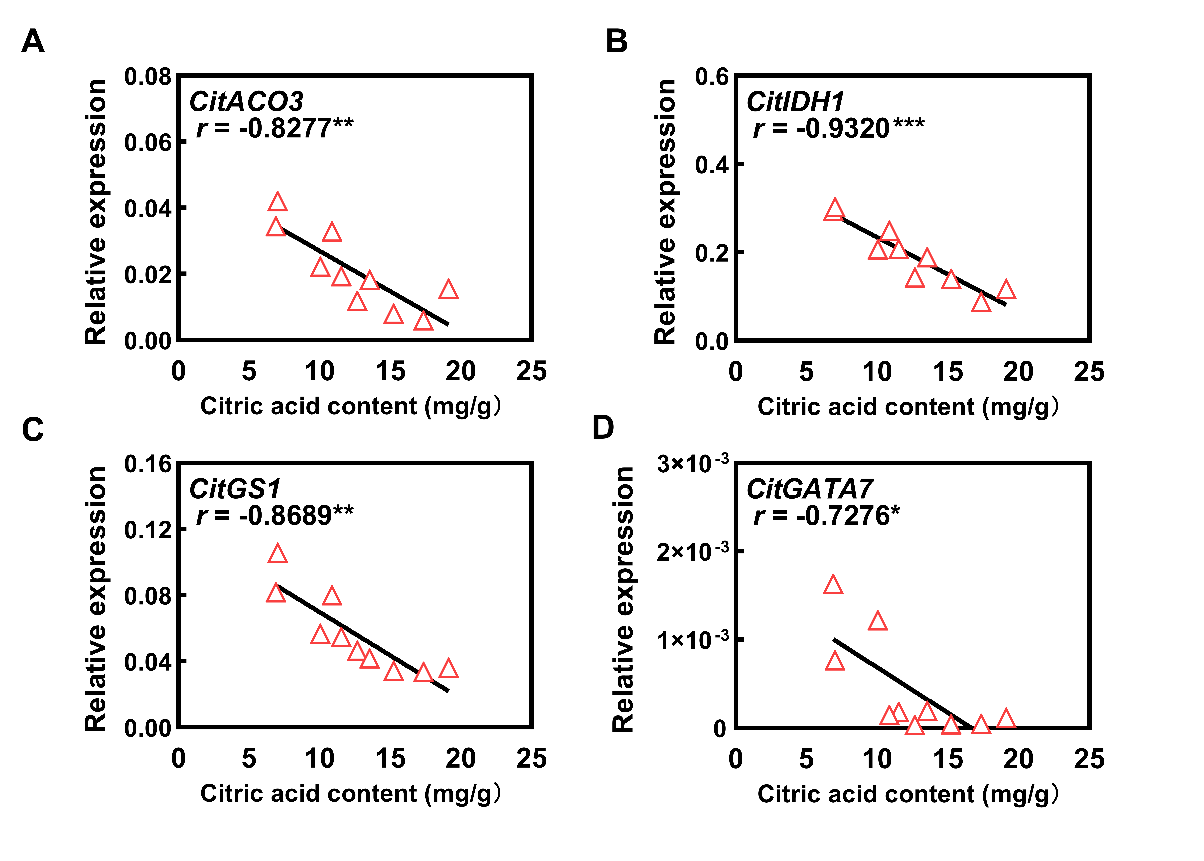


**Fig. S2** Correlation analysis between citric acid content and gene expressions. *CitACO3* (A**)**, *CitIDH1* (B**)**, *CitGS1* (C**)**, and *CitGATA7* (D**)***.* Statistical significance was determined by two-tailed test (**p <* 0.05, ***p <* 0.01, ****p <* 0.001).


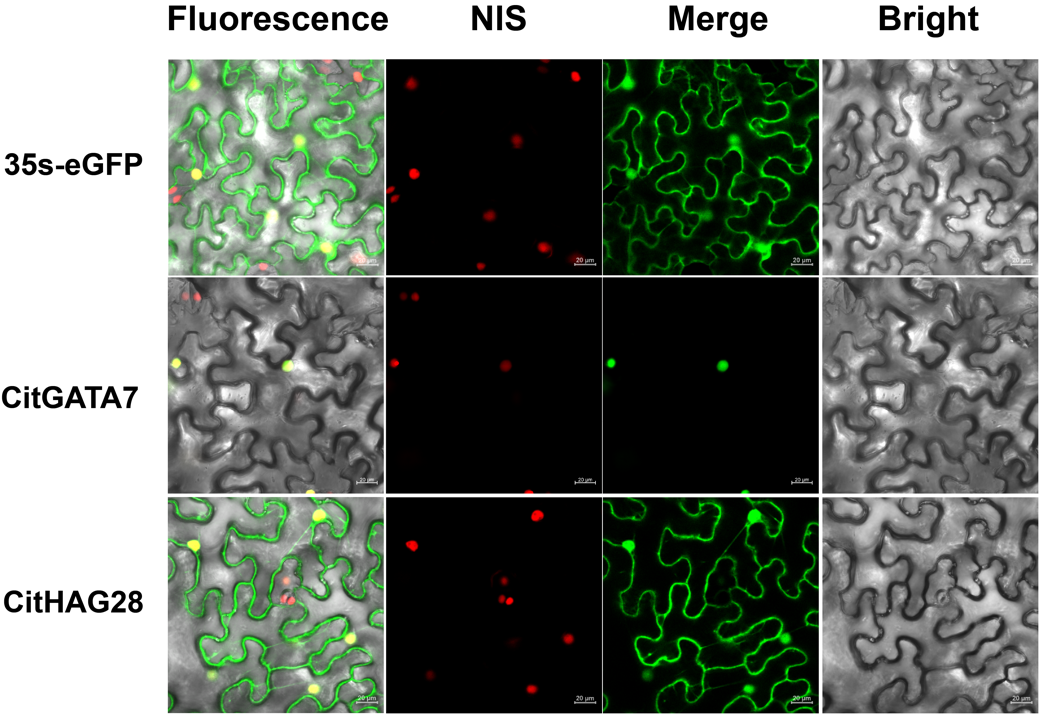


**Fig. S3** Subcellular localization analysis of CitGATA7 and CitHAG28 in transgenic *Nicotiana benthamiana* leaves. 35s-eGFP serves as a positive control. Bar = 20 μm.

**
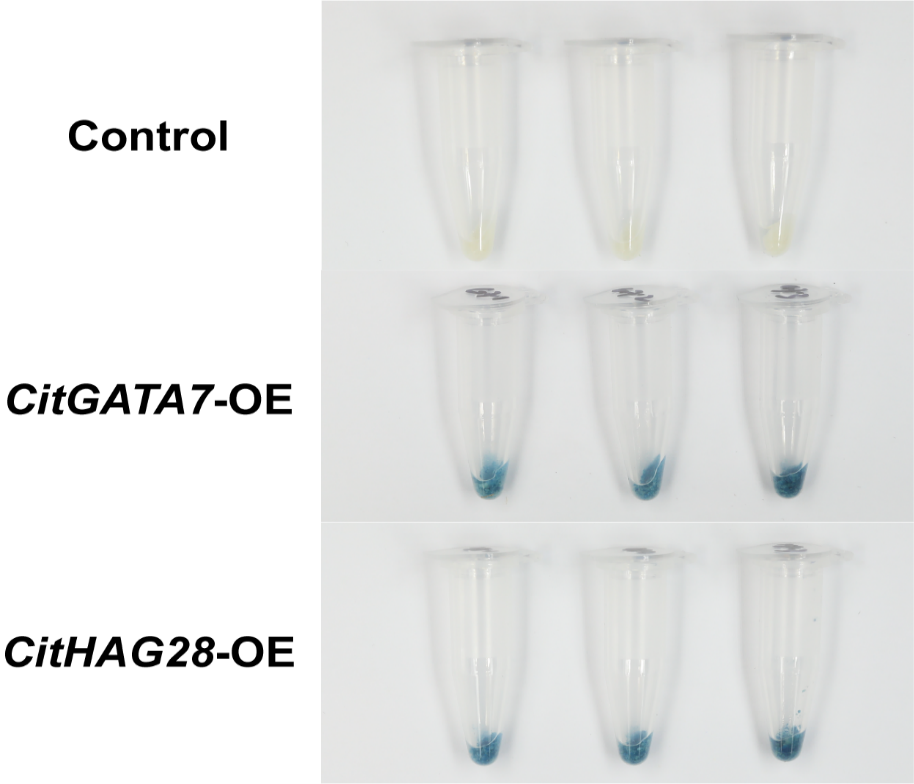
**

**Fig. S4** GUS staining analysis in citrus callus of the control, *CitGATA7*-OE and *CitHAG28*-OE*.* The blue coloration indicates positive citrus callus successfully transformed with the pCAMBIA1301 vector.


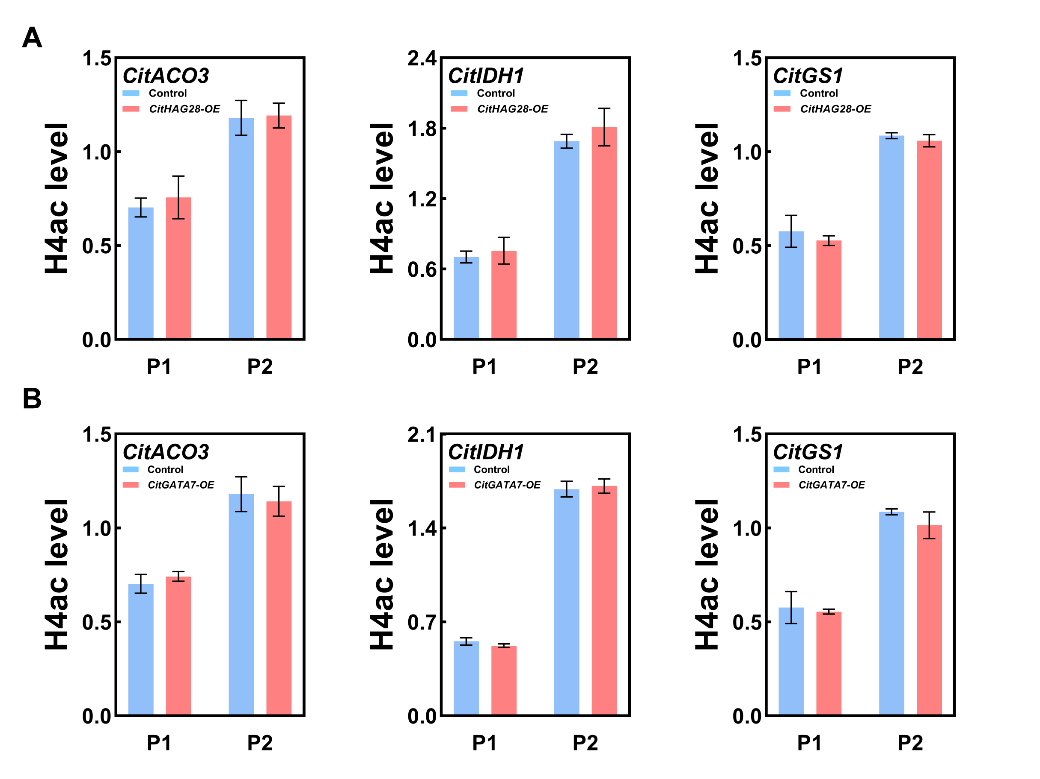


**Fig. S5** The level of H4ac around the promoter (P1) and near the TSS (P2) of *CitACO3*, *CitIDH1*, and *CitGS1*. (A) *CitHAG28*-OE citrus callus; (B) *CitGATA7*-OE citrus callus. Error bars indicate the standard error (SE) (*n* = 3).
